# Microbial Interactions in Kombucha through the Lens of Metabolomics

**DOI:** 10.3390/metabo12030235

**Published:** 2022-03-09

**Authors:** Thierry Tran, Chloé Roullier-Gall, François Verdier, Antoine Martin, Philippe Schmitt-Kopplin, Hervé Alexandre, Cosette Grandvalet, Raphaëlle Tourdot-Maréchal

**Affiliations:** 1 UMR Procédés Alimentaires et Microbiologiques, Institut Agro Dijon, Université de Bourgogne Franche-Comté, 21000 Dijon, France; chloe.roullier-gall@u-bourgogne.fr (C.R.-G.); rvalex@u-bourgogne.fr (H.A.); cosette.grandvalet@u-bourgogne.fr (C.G.); tourdot@u-bourgogne.fr (R.T.-M.); 2 Biomère, 14 rue Audubon, 75120 Paris, France; fverdier@jubiles.bio (F.V.); amartin@jubiles.bio (A.M.); 3 Comprehensive Foodomics Platform, Technische Universität München, 85354 Freising, Germany; schmitt-kopplin@helmholtz-muenchen.de; 4 Research Unit Analytical BioGeoChemistry, Department of Environmental Sciences, Helmholtz Zentrum München, 85764 Neuherberg, Germany

**Keywords:** kombucha, interaction, metabolomics, process, yeast, acetic acid bacteria, fermentation

## Abstract

Kombucha is a fermented beverage obtained through the activity of a complex microbial community of yeasts and bacteria. Exo-metabolomes of kombucha microorganisms were analyzed using FT-ICR-MS to investigate their interactions. A simplified set of microorganisms including two yeasts (*Brettanomyces bruxellensis* and *Hanseniaspora valbyensis*) and one acetic acid bacterium (*Acetobacter indonesiensis*) was used to investigate yeast–yeast and yeast–acetic acid bacterium interactions. A yeast–yeast interaction was characterized by the release and consumption of fatty acids and peptides, possibly in relationship to commensalism. A yeast–acetic acid bacterium interaction was different depending on yeast species. With *B. bruxellensis*, fatty acids and peptides were mainly produced along with consumption of sucrose, fatty acids and polysaccharides. In opposition, the presence of *H. valbyensis* induced mainly the decrease of polyphenols, peptides, fatty acids, phenolic acids and putative isopropyl malate and phenylpyruvate and few formulae have been produced. With all three microorganisms, the formulae involved with the yeast–yeast interactions were consumed or not produced in the presence of *A. indonesiensis*. The impact of the yeasts’ presence on *A. indonesiensis* was consistent regardless of the yeast species with a commensal consumption of compounds associated to the acetic acid bacterium by yeasts. In detail, hydroxystearate from yeasts and dehydroquinate from *A. indonesiensis* were potentially consumed in all cases of yeast(s)–acetic acid bacterium pairing, highlighting mutualistic behavior.

## 1. Introduction

As the knowledge linking human microbiota (especially in the gut) to human health deepens, the interest regarding the consumption of fermented food with or without live microorganisms (dietary microorganisms) is increasing [1,2]. Evidence pointing the benefits of dietary microorganisms and their activity on the nutritional quality of food accumulates as new strong hypotheses emerged on the interactions of live foodborne microorganisms and their metabolites with the gut microbiota [3]. Therefore, the study of microbial interactions is relevant to understand phenomena and mechanisms that occur in the context of food fermentations, whether those interactions involve foodborne microorganisms and the host, or the fermenting microorganisms themselves in relationship to the control of fermentations involving complex microbial communities [4].

Kombucha is a traditional beverage of interest regarding the consumption of dietary microorganisms. It is produced through the transformation of sugared tea infusion by a consortium of yeasts and acetic acid bacteria, with or without the presence of lactic acid bacteria [5]. A recent study determined that *Komagataeibacter* was the dominant genus for bacteria and *Brettanomyces bruxellensis* was the dominant species of yeasts [6]. However, other genera can be found instead or along them such as *Acetobacter*, *Gluconacetobacter* and *Liquorilactobacillus* for bacteria and *Hanseniaspora*, *Saccharomyces*, *Picha,* or *Zygosaccharomyces* for yeasts [6,7,8]. It has been determined that those consortia (occurring with many different compositions in genera and species) used as starter cultures induced different microbial interactions. On the metabolic level, acetic acid bacteria are dependent on yeasts for the release of assimilable nutrients that are glucose, fructose and ethanol released through invertase activity then alcoholic fermentation [9]. Microbial interactions in foods were traditionally studied through the use of culture-dependent microbiological analysis and targeted chemical analyses as it was the case in sourdough [10]. However, the growing accessibility of “omics” approaches involving non culture-dependent microbiological analyses (metagenomics) and nontargeted chemical analysis (metabolomics, proteomics) opened new perspectives for the study of food matrices and fermentations [11]. Therefore, the use of other approaches and analytic tools, namely non-targeted ones, could help to uncover other types of interactions in kombucha [5,8].

Microbial interactions can be categorized according to positive or negative impact of one microorganism population on another [12]. Competition for resources can occur between population sharing similar needs in nutrients. Amensalism refers to a negative impact of one population on another, for example through the secretion of toxins. Commensalism corresponds to the production of one metabolite by one population that can be exploited by another. In the case that one population benefits from another, while impacting the later negatively, the interaction is characterized by parasitism. Finally, if both population benefits from each other’s presence, it is characterized by mutualism.

Fourier transform ion cyclotron resonance mass spectrometry (FT-ICR-MS) is a non-targeted analysis of nonvolatile compounds that has already been used to study microbial interaction using metabolomics in fermented beverages, particularly grape must in the context of wine fermentation. Metabolites present in the food matrix, including those released by microorganisms, are named as a whole “exo-metabolome” and the qualitative study of exo-metabolomes is called “footprinting” [13]. Interspecies yeast–yeast interactions between *Saccharomyces* and non-*Saccharomyces* species in grape must have been investigated by comparing exo-metabolomes obtained by FT-ICR-MS [14,15]. These studies confirmed the impact of such interaction on the composition in metabolites and namely the effect of cell-cell contact on yeast metabolism which involved compounds from different molecular families (carbohydrates, amino acids, nucleic acids, and polyphenols). Interkingdom yeasts-bacteria interactions have also been investigated between *Saccharomyces cerevisiae* and *Oenococcus oeni* following a similar approach [16]. Detailed interpretation of data highlighted individual markers such as gluconic acid, citric acid, trehalose and palmitic acid, which were reported to be metabolized by both microorganisms and be the support of metabolic interplay.

The present study aims at analyzing and comparing exo-metabolomes of monocultures and cocultures in sugared tea of microorganisms isolated from the same kombucha consortium using FT-ICR-MS tool. The study involved two yeasts (*B. bruxellensis* and *Hanseniaspora valbyensis*) and one acetic acid bacterium (*Acetobacter* *indonesiensis*) that are representative of the microbial composition of the original kombucha consortium in terms of population levels. Additionally, those genera and species have been reported to be widely represented in kombucha consortia in general and sucrose-based fermented food [6,11]. The qualitative and quantitative comparison of exo-metabolomes is expected to highlight the stimulation or inhibition of non-volatile metabolites release or pathways associated with microbial interactions between yeasts and the acetic acid bacterium.

## 2. Results and Discussion

### 2.1. Comparison of General Chemical Compositions and Data Visualization

**Figure 1:**
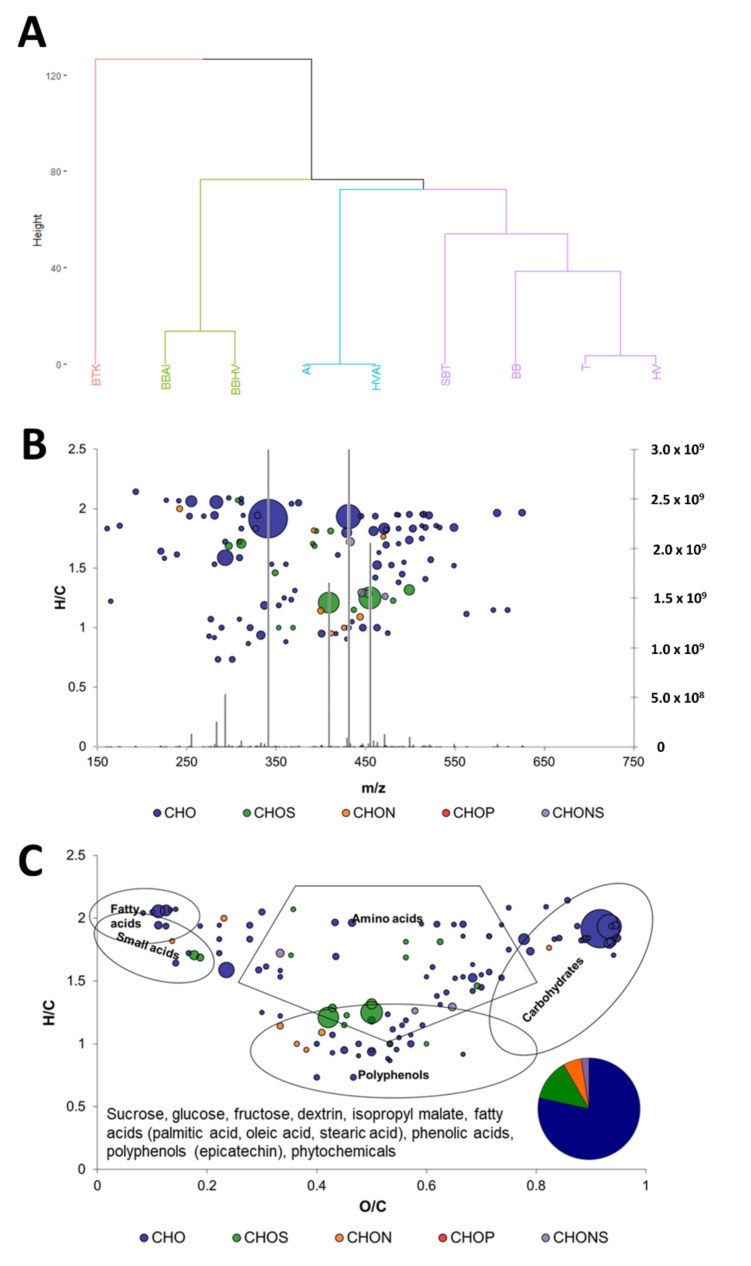
Visualization of composition differences between the samples analyzed by FT-ICR/MS (**A**) HCA dendrogram showing composition similarities between sugared black tea (SBT), cultures at day 7 including *Brettanomyces bruxellensis* (BB), *Hanseniaspora valbyensis* (HV), and *Acetobacter indonesiensis* (AI) isolated from black tea kombucha (BTK), structured after hierarchical clustering. (**B**) Superposition of mass spectrum and distribution of annotated element formula according to H/C ratio and *m*/*z*. Colors distinguish different elemental compositions. (**C**) van Krevelen diagram of common formulae across Sugared Black Tea and all cultures (“core metabolites” group), complemented with the proportions in different elemental compositions. The utilization of van Krevelen diagrams allows the determination of regions corresponding to chemical families such as lipids, small acids, amino acids, polyphenols or carbohydrates, based on the analysis of a large number of compounds [17]. The surface of bubbles expresses relative formulae’s intensity. Comparison of mass lists with databased such as METLIN, putative compound identities can be obtained. Most probable candidates were added in the bottom left-hand corner of the diagram.

Peak intensities associated to ion masses detected in sugared tea and cultures samples were analyzed using hierarchical clustering analysis (HCA) after data treatment to display similarity in non-volatile chemical compositions (Figure 1A). Black tea kombucha (BTK) has been included to assess the closeness in composition of cultures from isolated kombucha microorganisms with original kombucha. Clusters separated mainly BTK samples from those cultures and sugared black tea (SBT), highlighting a strong distance between original kombucha and the cultures in terms of nonvolatile chemical composition. This result can be explained by the difference in microbial compositions with BTK possessing much higher microbial diversity than the other cultures [9]. Moreover, the inoculation methods were different, with the inclusion of metabolites from the previous batch in the case of BTK corresponding to traditional kombucha production method. This did not happen for the other cultures that occurred through the addition of washed cells grown on culture medium. Indeed, those cultures constitute model systems as opposed to the original kombucha which is used as a reference in the present study. Inside the cluster excluding BTK, the main subclusters discriminated cocultures involving *B. bruxellensis* except the coculture T with the simultaneous presence of the three microorganisms. The yeast *B. bruxellensis* appeared to strongly impact the composition of the medium in association with *H. valbyensis* only or with *A. indonesiensis* only. In the remaining branch, AI and HVAI were opposed to SBT, BB, HV, and T. It appears that the presence of *H. valbyensis* in HVAI coculture had little impact when compared to the AI monoculture, suggesting an important impact of *A. indonesiensis*. Also, it seems that yeasts (BB and HV) induced the least changes of the matrix SBT in comparison with the acetic acid bacterium in monoculture since they belonged to the same cluster. The presence of the coculture of the two yeasts and one acetic acid bacterium T in this cluster was surprising, because of its apparent dissimilarity with other cocultures (BBAI, HVAI, BBHV) that are closer to this condition on the microbiological level. Additionally, with a higher biodiversity, T could be expected to not share the same cluster than the unfermented matrix SBT. It is worth noting that yeasts monocultures were closer than the acetic acid bacterium monoculture, highlighting proximity between organisms of the same kingdom. Detailed study of metabolite transformations will help understanding the reason of such clustering. A similar approach has been used in the context of wine fermentation using different pairings of *Saccharomyces cerevisiae* with non-*Saccharomyces* species: *Lachancea thermotolerans*, *Metschnikowia pulcherrima,* or *Starmerella bacillaris*. FT-ICR-MS analysis resulted in clustering depending on the presence of the non-*Saccharomyces* species, except for *L. thermotolerans*, thus highlighting interactive effects as well [15]. However, several elements of data visualization need to be explained beforehand.

Direct infusion FT-ICR-MS was used for metabolite profiling, enabling a wide dynamic range in intensity (10^6^) and we focused on the most abundant compounds (S/N > 4). After data processing, peaks appearing on FT-ICR-MS mass spectra associate a set of ion masses (*m*/*z*) with intensity, thus providing semi-quantitative data (Figure 1B). Based on highly precise measured *m*/*z* values, each value was annotated with a molecular or elemental formula in term of composition carbon (C), hydrogen (H), oxygen (O), nitrogen (N), and sulfur (S) atoms, which distinguishes FT-ICR-MS from other tools for metabolomics. The formulae obtained in the CHONS-space can be visually represented with different colors based on their elemental compositions and spatially using elemental ratios, for example H/C. The utilization of H/C and O/C ratios corresponds to the van Krevelen diagram and it can be complemented with a diagram of the proportion in elemental compositions. Figure 1C represents the 136 formulae common to all samples. This group was labeled as “core metabolites” in a van Krevelen diagram. This representation gives more information on the formulae, since it allowed the determination of zones corresponding to chemical families such as lipids, amino acids, carbohydrates, and polyphenols [17]. Moreover, relative intensities can be expressed through the surface of the bubbles associated to each formula. The core metabolites group included mainly CHO formulae and CHOS and CHON in lesser proportion. Different chemical families were represented (lipids, amino acids, carbohydrates, and polyphenols). This core metabolome is very different from those of grape must and wine matrices, which are richer in amino acids, carbohydrates, and nucleic acids. In contrast, high diversity in polyphenols and majority of CHO elementary formulae are common with the SBT matrix [14]. By comparing the masses with databases, such as METLIN used in the present study, it is possible to annotate them with putative identities (level 3 annotation; Table S1) [18,19]. In the case of the core metabolites group, putative identities from the sugared tea matrix could be obtained, like sucrose, glucose, fructose, dextrin, isopropyl malate (intermediate of leucine biosynthesis), palmitic, stearic, and oleic acids, along with various polyphenols and phytochemicals (plant compounds).

Additionally, coupled utilization of MASSTRIX application [20] for annotation with KEGG Mapper Color application allowed to associate putative identities with molecular pathways. Annotation was performed using specific databases for *Saccharomyces cerevisiae* (model yeast) and *Acetobacter pasteurianus* (acetic acid bacterium). Distribution of putative identities according to metabolic pathways is available in Figure S2. Common pathways, such as sugar metabolism or the biosynthesis of cofactors were mainly represented. Pathways exclusive to yeasts involve starch and sucrose metabolism, and alpha linoleic acid metabolism, whereas only aromatic compounds degradation pathways were specific to the acetic acid bacterium.

### 2.2. Metabolic Signature of Individual Microorganisms

**Figure 2:**
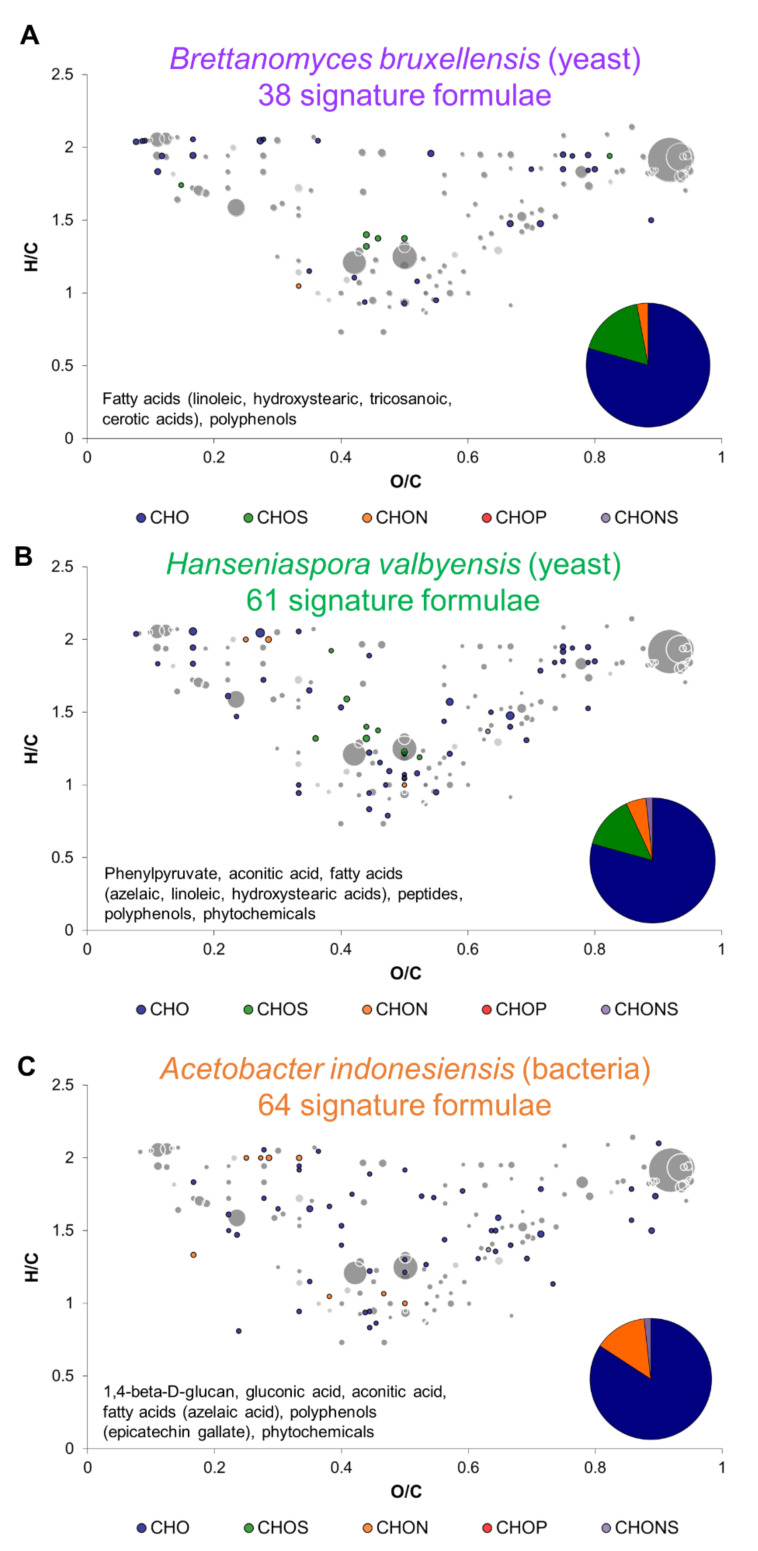
van Krevelen diagrams of formulae that are not common to all three microorganisms (signature formulae): (**A**) *Brettanomyces bruxellensis*, (**B**) *Hanseniaspora valbyensis,* and (**C**) *Acetobacter indonesiensis*, with proportions in elemental compositions and putative identities. Core metabolites formulae (Figure 1C) are represented in the background in grey.

Before comparing monocultures and cocultures exo-metabolomes, signature formulae of each microorganism studied were represented in van Krevelen diagrams (Figure 2). Signature formulae were associated with one of the microorganisms if they were detected significantly in higher intensity than one of the two other microbes. *H. valbyensis* and *A. indonesiensis* possessed a similar number of signature formulae (61 and 64 respectively), whereas *B. bruxellensis* had less (38). The signature formulae of all three microorganisms were composed mostly of CHO. For yeasts, they were composed to a lesser extent of CHOS and CHON, in opposition with the signature formulae of *A. indonesiensis* that possessed no CHOS. Signature formulae also belonged to different molecular families: mainly fatty acids, carbohydrates, and polyphenols. Plant metabolites, polyphenols and phytochemicals, were molecular families that widely discriminated the microorganisms. It is worth noting that epicatechin gallate was potentially associated to *A. indonesiensis* and was reported in kombucha by several studies (Figure 2C) [21,22]. Additionally, *B. bruxellensis* and *H. valbyensis* distinguished themselves from the acetic acid bacterium with fatty acids (potentially linoleic and hydroxy stearic acids; Figure 2A,B). *H. valbyensis* and *A. indonesiensis* shared azelaic acid and aconitic acid (intermediate between citrate and isocitrate in the TCA cycle) as putative signature compounds (Figure 2B,C). However, the putative compound phenylpyruvate was specific to *H. valbyensis*. This metabolite is a precursor of the volatile organoleptic compounds phenylethanol and phenylethyl acetate, which have been reported to be produced in higher amounts by this yeast species in both kombucha and cider [23]. The fact that peptides were also specific of this yeast species could be linked to the fact that it experienced more mortality inducing the release of intracellular peptides. Indeed, the genome of this species informs that it is part of a fast-evolving lineage that lost genes related to DNA repair and cell cycle that could induce higher mortality [24]. Regarding *A. indonesiensis*, gluconic acid appeared to be a signature compound. This metabolite results from the oxidation of glucose [25]. Another signature formula was associated to 1,4-β-D-glucan putative identity, probably cellulose which shares the same formula. Cellulose is also a typical product of acetic acid bacteria leading to the formation of kombucha pellicle through the utilization of monosaccharides (glucose and fructose) [7,26]. Globally, metabolic signatures differentiated microorganisms, in relation with their kingdom. The next step examined the changes brought by the interaction of those microorganisms when cultured together in sugared black tea.

### 2.3. Yeast–Yeast Interspecies Interactions

**Figure 3:**
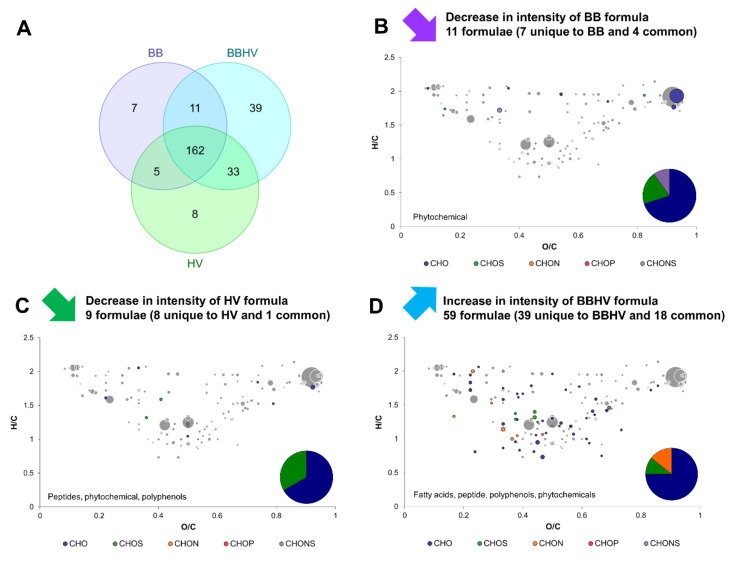
Change in composition induced by the yeast–yeast interaction of *B. bruxellensis* (BB) and *H. valbyensis* (HV). (**A**) Venn diagram of formulae identified in BB and HV monocultures and the BBHV coculture. van Krevelen diagrams and putative identities of formula (**B**) decreasing in BB monoculture, (**C**) decreasing in HV monoculture and (**D**) increasing in BBHV coculture. Core metabolites formulae (Figure 1C) are represented in the background in grey.

Exo-metabolomes of *B. bruxellensis* and *H. valbyensis* in separate monocultures in sugared tea (BB and HV) were compared to the exo-metabolome of both yeasts in coculture (BBHV). Figure 3A shows that most of metabolites were detected in all conditions (162 over 265) and that 39 new formulae were detected exclusively in the coculture. In contrast, fewer formulae present in monoculture were not detected in the coculture (20 in total). Only 11 formulae underwent a significant decrease in intensity for BB and were CHO in majority, along with CHOS and CHONS. The same observation occurred for HV with 9 formulae. According to putative compound annotations, phytochemicals and polyphenols were involved. The decrease in *H. valbyensis* peptides intensities could be due to their consumption by *B. bruxellensis* in the coculture, which would characterize a case of commensalism. In turn, 59 formulae significantly increased in intensity because of the coculture, with a majority of CHO, CHOS, and CHON. Annotation with putative identities implied the increase of fatty acids, one peptide, polyphenols and phytochemicals. These results were similar to those obtained in the context of grape must fermentation when pairing two different yeasts species. When *S. cerevisiae* was paired with *L. thermotolerans* or *S. bacillaris*, the number of newly produced formulae including amino acids, polyphenols and carbohydrates, was higher in the coculture than in the monoculture of *S. cerevisiae*. This was the opposite when paired with *M. pulcherrima*, thus highlighting a species factor and not a general phenomenon [15]. Overall, yeast–yeast interaction between *B. bruxellensis* and *H. valbyensis* influenced microbial metabolism by inducing the release or consumption of fatty acids and peptides, possibly in relationship to commensalism. Moreover, it impacted the structure of tea compounds.

### 2.4. Yeast–Acetic Acid Bacterium Interactions

**Figure 4:**
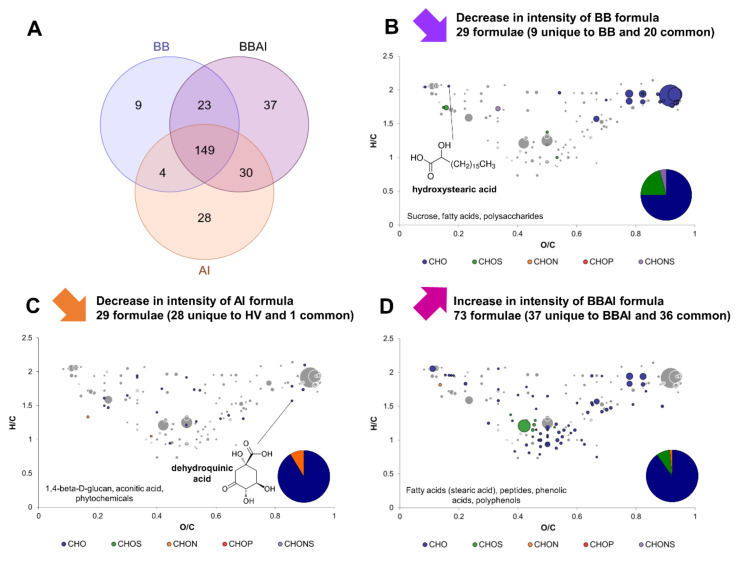
Change in composition induced by the yeast–acetic acid bacteria interaction of *B. bruxellensis* (BB) and *A. indonesiensis* (AI). (**A**) Venn diagram of formulae identified in BB and AI monocultures and the BBAI coculture. van Krevelen diagrams and putative identities of formulae (**B**) decreasing in BB monoculture, (**C**) decreasing in AI monoculture and (**D**) increasing in BBAI coculture. Core metabolites formulae (Figure 1C) are represented in the background in grey.

**Figure 5:**
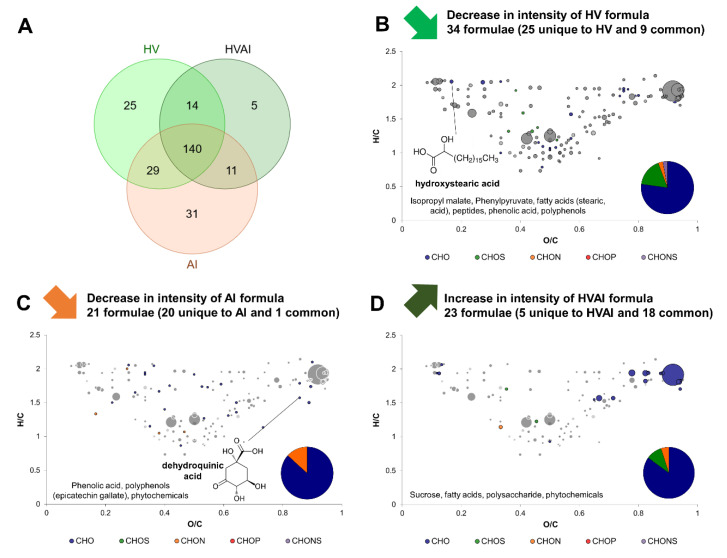
Change in composition induced by the yeast–acetic acid bacteria interaction of *H. valbyensis* (HV) and *A. indonesiensis* (AI). (**A**) Venn diagram of formulae identified in HV and AI monocultures and the HVAI coculture. van Krevelen diagrams and putative identities of formulae (**B**) decreasing in HV monoculture, (**C**) decreasing in AI monoculture and (**D**) increasing in HVAI coculture. Core metabolites formulae (Figure 1C) are represented in the background in grey.

Exo-metabolomes of *B. bruxellensis* and *A. indonesiensis* in separate monocultures (BB and AI) were compared to the exo-metabolome of both microorganisms in coculture (BBAI). The same was applied with the yeast *H. valbyensis* in association with the cocultures (HVAI). In both cases, most of formulae were detected in all conditions (140 over 280 and 149 over 255 for the comparisons involving *B. bruxellensis* and *H. valbyensis* respectively; Figure 4A and Figure 5A). Based on the Venn diagrams, two very distinct behaviors could be observed. BBAI coculture induced the production of 37 formulae and the disappearance of 28 formulae unique to AI monoculture, and 9 formulae unique to BB (Figure 4A). In contrast, the HVAI coculture only produced 5 new and unique formulae. The number of formulae unique to AI monoculture was 31 in similar amount as those affected by the BBAI coculture. However, 25 formulae were unique to HV, which is more than those of BB (Figure 5A).

CHO represented the majority of yeast formulae that underwent a decrease in intensity, along with CHOS and CHONS. CHON were only detected for HV monoculture (Figure 4B and Figure 5B). Among formulae annotated with putative identities, only hydroxy stearic acid underwent a decrease in intensity in both cases. Sucrose was significantly more consumed in BBAI than BB along with other fatty acids and polysaccharides. Greater sucrose consumption could be explained by the adequation of *B. bruxellensis* and *A. indonesiensis* cocultures, leading to efficient sugar consumption and production of organic acids in the context of kombucha production [9]. *B. bruxellensis* was reported to have higher invertase and fermentative activities allowing better accessibility of substrate for acetic acid bacteria [9]. In contrast, HV formulae that underwent a decrease in intensity due to HVAI coculture were polyphenols and metabolites that could be involved in commensalism interactions: peptides, fatty acids, phenolic acids, isopropyl malate and phenylpyruvate, that are involved respectively in leucine and phenylalanine metabolism and widely useable by other organisms [27].

When *A. indonesiensis* was paired with a yeast, a majority of CHO compounds and some CHON formulae (no CHOS or CHONS) underwent a decrease in intensity (Figure 4C and Figure 5C). Putative identities involved phytochemicals and dehydroquinic acid (an intermediate in the shikimate pathway) regardless of the yeast in coculture. In coculture with *B. bruxellensis*, a decrease in intensity of putative aconitic acid and 1,4-β-D-glucan could be observed, whereas phenolic acids and polyphenols including epicatechin gallate appeared to be impacted in the presence of *H. valbyensis*. In the first case, carbon metabolism of *A. indonesiensis* seemed to be influenced, whereas secondary plant metabolisms were transformed on the second case.

The formulae significantly produced in coculture were mainly CHO and CHON or CHOS to a lesser extent (Figure 4D and Figure 5D). They included fatty acids in both cases, highlighting an effect on microbial metabolism [28,29]. In the case of BBAI, stearic acid was specifically released as well as peptides, possibly due to yeast autolysis. Additionally, phenolic acids could have been converted and polyphenols were modified. In the case of HVAI, less sucrose was consumed and polysaccharides were produced, whereas phytochemicals were affected.

Overall, yeast–acetic acid bacterium interactions were substantially different depending on the yeast species, with apparent effects on compounds from different metabolic pathways. The interaction of *A. indonesiensis* with *B. bruxellensis* seemed to affect the exo-metabolome towards higher molecular diversity through the stimulation of carbon metabolism, while the interaction with *H. valbyensis* led to a lower molecular diversity through the consumption of *H. valbyensis* metabolites. In all cases however, tea compounds were affected by microbial interactions.

### 2.5. Complex Interactions Involving Yeast–Yeast and Yeast–Acetic Acid Bacterium Interaction

**Figure 6:**
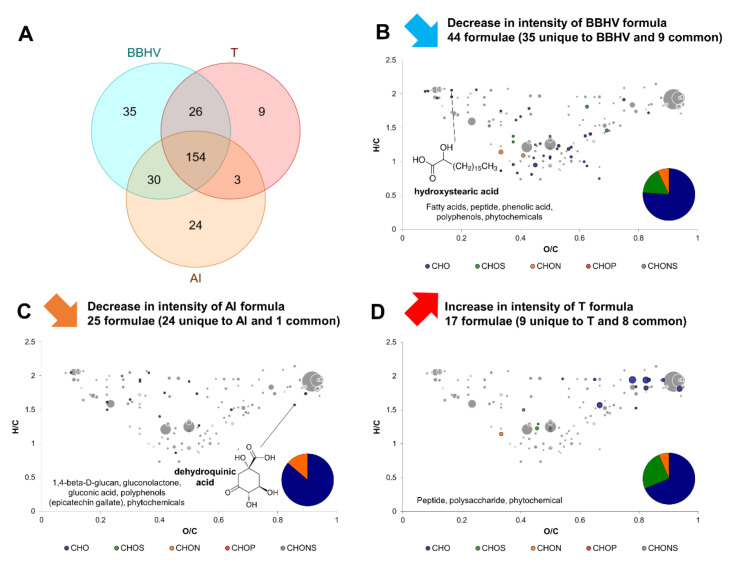
Change in composition induced by the complex yeast–acetic acid bacteria interaction of *B. bruxellensis* and *H. valbyensis* coculture (BBHV) and *A. indonesiensis* (AI). (**A**) Venn diagram of formulas identified in BBHV and the T coculture including the two yeasts and the acetic acid bacteria. van Krevelen diagrams and putative identities of formulae (**B**) decreasing in BBHV coculture, (**C**) decreasing in AI monoculture and (**D**) increasing in T coculture. Core metabolites formulae (Figure 1C) are represented in the background in grey.

Exo-metabolomes of *B. bruxellensis* and *H. valbyensis* yeast–yeast coculture (BBHV) and *A. indonesiensis* in monoculture (AI) were compared to the exo-metabolome of the coculture gathering the three microorganisms (T). Similarly with previous comparisons, a big part of formulae was detected in all conditions (154 over 281 in total; Figure 6A). The Venn diagram shows that only 9 formulae were unique to T, while 35 and 24 unique formulae from BBHV and AI respectively (plus 30 common formulae) were not detected in T. This case was similar to the interaction between *H. valbyensis* and *A. indonesiensis* with a loss in molecular diversity due to the interaction between the yeast and the bacterium. 44 formulae from BBHV underwent a decrease in intensity and included mainly CHO, CHON and CHOS in lesser amounts (Figure 6B). Very diverse molecular families were impacted. According to putative identity annotations, these families included fatty acids, hydroxy stearic acid, a peptide, a phenolic acid, polyphenols and phytochemicals. Twenty-five formulae from AI underwent a decrease in intensity, with similar amounts compared to the BBAI and HVAI interactions (28 and 31, respectively). These formulae were mainly represented by CHO, with a smaller portion of CHON. No CHOS was detected. Annotations with putative identities suggested a decrease in intensity of the same compounds as for BBAI: 1,4-βD-glucan (or cellulose), dehydroquinic acid, gluconic acid and epicatechin gallate, along with gluconolactone, other polyphenols and phytochemicals. Finally, 17 formulae underwent an increase in intensity in T composed of CHO, CHOS and CHON, including potentially a peptide, a polysaccharide and a phytochemical. The result of this complex interaction of two yeasts and one acetic acid bacterium is surprising because of the loss of molecular diversity, when microbial interactions rather stimulate the production of compounds in reaction to the presence of other microorganisms [15]. This was namely the case for the yeast–yeast interaction between *B. bruxellensis* and *H. valbyensis*.

The formulae unique to BBHV produced by the interaction of the two yeasts were compared with the formulae unique to BBHV that where not detected in the presence of *A. indonesiensis* in T (Figure S3). It appears that 30 formulae out of 57 that were produced from the yeast–yeast interaction were inhibited by the presence of *A. indonesiensis*. It may be assumed that the yeast–acetic acid bacterium interaction had either consumed or prevented the production of the metabolites produced by the yeast–yeast interaction. Potential metabolite identities include few fatty acids, phenolic acids, polyphenols and phytochemicals. Therefore, it is not clear if metabolites, potentially signaling molecules could be consumed by *A. indonesiensis* as part of commensalism. However, the interaction of microbial activity with polyphenols was clearly inhibited in the presence of the bacterium. The mechanism behind this phenomenon remains unclear, but it can be stated that the transformation underwent by tea compounds were dependent on microbial activity with variations according to the species. Modification of phenolic compounds during kombucha production have been reported in a recent study using metabolomics [30]. However, specific metabolic pathways including polyphenols have not been characterized in kombucha species. Such enzymatic activity of polyphenol conversion have been identified in the context of the gut microbiota [31].

Regarding the effects of yeast–acetic acid bacterium interaction, formula associated to putative hydroxystearic acid from yeast cultures (BB, HV and BBHV) decreased systematically in intensity in the presence of *A. indonesiensis.* The position of hydroxy stearic acid is indicated on van Krevelen diagrams on Figure 3B, Figure 4B, Figure 5B, and Figure 6B. This mono-unsaturated fatty acid was reported as the intermediate between stearic acid and oleic acid. This reaction was thought to be performed by *Saccharomyces cerevisiae* for a long time. A recent study suggested that it was instead caused by bacterial contaminants, in contradiction with our results with an apparent hydroxy stearic production by *B. bruxellensis* and *H. valbyensis* [32]. However, the decrease in intensity observed in the presence of *A. indonesiensis* could be explained by the conversion of hydroxy stearic acid into volatile organoleptic γ-dodecalactone, which has been reported for bacteria, and namely lactic acid bacteria in the context of whisky production [33,34]. As supporting elements, acetic acid bacteria have been reported to convert 1,4-nonanediol into γ-nonanoic lactone [35] and gluconolactone was detected as a putative compound in our study. This metabolite acts as an intermediate in the oxidation of glucose into gluconic acid by *Acetobacter senegalensis* according to KEGG database [36]. Consequently, the decrease in hydroxystearic acid, which is strongly associated to yeast-bacteria interactions in the literature, could be explained by a conversion performed by the acetic acid bacterium present, as part of a commensalism.

Moreover, a pattern regarding the formulae associated to AI negatively affected by yeast–acetic acid bacterium interaction could be observed. The number of those compounds was consistent in each case (between 24 and 31) and consisted in CHO and CHON exclusively, with no CHOS detected. The lists of AI formulae that were negatively impacted by the presence of yeast across the three cases of yeast–acetic acid bacterium interaction were compared using a Venn diagram (BBAI, HVAI, and T; Figure S4). A total of 14 formulae out of 37 systematically underwent a decrease in intensity in the presence of yeasts in all three cases, and other 14 formulae did in 2 cases out of 3. This suggests that the impact of the yeasts’ presence on *A. indonesiensis* was consistent regardless of the yeast species. In opposition, the impact of the acetic acid bacterium on the yeast formulae differed significantly according to the yeast species. The putative compound identities involve polyphenols, phytochemicals, fatty acids, and dehydroquinic acid. This last metabolite is a key intermediate of the shikimate pathway that connects glycolysis to the biosynthesis of aromatic amino acids. The decrease in the presence of yeasts could suggest a case of commensalism if it is internalized by yeast to supply their own production of aromatic amino acids [27]. Moreover, the presence of dehydroquinic acid in the medium can be explained by the presence of quinate dehydrogenase enzyme (converting quinic acid into dehydroquinic acid) on the membrane of acetic acid bacteria [37]. The position of dehydroquinic acid is indicated on van Krevelen diagrams on Figure 3C, Figure 4C, Figure 5C, and Figure 6C. By providing such a key metabolic intermediate directly in the medium, aceticacid bacteria clearly favor commensalism regarding this compound. When put in perspective with their non-strict parasitic interaction towards yeast reported in the context of kombucha production [9], this element could advocate for a mutualistic interaction between yeasts and acetic acid bacteria.

## 3. Materials and Methods

### 3.1. Generation of Biological Samples

#### 3.1.1. Production of Original Black Tea Kombucha

Samples analyzed in this study include sugared tea infusion before and after kombucha fermentation or microbial activity of inoculated microorganisms. These samples are the same as in our recently published study (Tran et al., 2022) [38]. Briefly, sugared black tea (SBT) infusion was produced by steeping 1 g L^−1^ of black tea (Pu’er Grade 1 TN4107) from Les Jardins de Gaia© (Wittisheim, France) in boiling water for one hour. After cooling down at room temperature, 50 g L^−1^ blond cane sugar (sucrose) from Ethiquable© (Fleurance, France) was dissolved. Then 12% (*v/v*) of 7 days kombucha was added. The mother culture used to produce this 7 days kombucha was obtained from Biomère (Paris, France). After inoculation, incubation occurred at 26 °C in static conditions during 7 days for the first phase of production in open vessel, that corresponds to a biological acidification [5]. Aluminum foil was loosely applied on the bottle neck to prevent contact with particles and insects. Samples obtained from original kombucha culture, labelled BTK, were used to compare global metabolic signature with cultures from isolated microorganisms. Those cultures were carried out by inoculating sugared black tea with microorganisms isolated from the same kombucha consortium than BTK samples.

#### 3.1.2. Isolation, Identification, and Selection of Strains

Isolation and selection process was described in detail in a previous study [9]. Briefly, yeasts and bacteria were isolated from the broth and the pellicle using differential agar media, such as Wallerstein Lab agar [39]. Identification of microorganisms was performed using biomolecular methods (26S and 16S PCR for yeasts and bacteria, respectively). Among the 6 species of yeasts (*B. bruxellensis*, *H. valbyensis*, *Hanseniaspora opuntiae*, *Pichia* aff. *Fermentans,* and *Galactomyces geotrichum*) and three species of acetic acid bacteria (*A. indonesiensis*, *Acetobacter papayae,* and *Komagataeibacter saccharivorans*) identified, selection of yeasts and acetic acid bacteria for further investigation was made based on their representativity and functionality in the context of kombucha production [9]. For this study, two yeasts, *B. bruxellensis* and *H. valbyensis*, and one acetic acid bacteria *A. indonesiensis* were selected to investigate yeast interspecies interactions and yeasts–acetic acid bacterium interkingdom interactions. This choices of yeasts were made because their invertase and fermentative activities were very different (high for *B. bruxellensis* and low for *H. valbyensis*). The use of one acetic acid bacterium was made because the impact of the species on the chemical composition was secondary compared to the yeasts. Among the acetic acid bacteria isolated from the original kombucha, *A. indonesiensis* was easier to cultivate compared to *K. saccharivorans* and was able to perform sufficient acidification, based on previous results [9].

#### 3.1.3. Cultures Conditions and Monitoring

Monocultures of *B. bruxellensis* (BB), *H. valbyensis* (HV), *A. indonesiensis* (AI), yeast–yeast coculture (BBHV), yeasts–acetic acid bacterium (BBAI, HVAI) and the trio made of two yeasts and one acetic acid bacterium (T) were produced. Cells washed with sugared black tea were inoculated at the rate of 1.0 10^5^ cells mL^−1^ using the same procedure as in our previous study [9]. Growth of microorganisms was monitored using culture-dependent methods as described in our previous study [38]. Each culture was performed in triplicates in 123 mL Boston flasks with a Specific Interfacial Surface (SIS) [40] of 0.01 cm^−1^. Sugared tea samples possessed a total sugar content of 58.9 ± 1.0 g L^−1^, a pH value of 6.90 ± 0.01 and a total acidity inferior to 1 meq L^−1^. Samples possessed a total sugar content ranging from 39.1 to 56.1 g L^−1^, pH values between 4.09 and 4.75 and a total acidity between 19 and 21 meq L^−1^. Important discrepancies in sugar contents, pH and total acidity values can be explained by very different activities between yeast and acetic acid bacteria monocultures compared to yeast–acetic acid bacteria cocultures, in which metabolic interplay is known to stimulate sugar metabolism and organic acid production [9]. Sugar content was measured using the Sucrose Glucose Fructose enzymatic kit from Biosentec (Portet-sur-Garonne, France). pH values were measured with a Mettler Toledo Five Easy pH meter coupled with a LE498 probe and total acidity was determined by titration with 0.1 N NaOH and 0.2% phenolphthalein as color indicator, with reagents purchased from Merck (Darmstadt, Germany).

### 3.2. Sample Preparation

Samples were centrifuged at 3000× *g* for 10 min at 4 °C to remove cells and particles before freezing at −18 °C. Once all samples were available, solid phase extraction (SPE) was performed using Bond Elut C18 cartridges from Agilent (Santa Clara, CA, USA). The aim of this step was to reduce the amount of sugar in the sample. Sugars are highly ionizable compounds that can then suppress the signal of other ion when present in large quantity [15]. Samples were acidified to reach a pH value between 1.5 and 2.0 using 50% (*v/v*) formic acid. The column was conditioned using 2 mL of methanol and 1 mL of 2% (*v/v*) formic acid. One mL of sample was added, followed by 1 mL of formic acid for washing. Extract was harvested by adding 1 mL of methanol. This extract was then diluted in methanol at the rate of 1/40 (*v/v*). Diluted samples were kept at −18 °C before analysis. All reagents were purchased from Fisher Scientific (Hampton, VA, USA). Formic acid (CAS 64-18-6) mother solution (50% (*v/v*) possessed puriss. p.a. grade and pure methanol (CAS 67-56-1) was UHPLC-MS grade with purity ≥99.9%.

### 3.3. Fourier Transform-Ion Cyclotron Resonance-Mass Spectrometry (FT-ICR-MS)

Ultrahigh-resolution FT-ICR-MS was performed with a 12 T Bruker solariX mass spectrometer (Bruker Daltonics, Bremen, Germany) equipped with an APOLLO II electrospray source in negative ionization mode [18]. The diluted samples were infused into the electrospray ion source at a flow rate of 120 μL h^−1^. Settings for the ion source were: drying gas temperature 180 °C, drying gas flow 4.0 L min^−1^, capillary voltage 3600 V. Spectra were externally calibrated by ion clusters of arginine (10 mg L^−1^ in methanol). Internal calibration of each spectrum was carried out using a reference list including selected markers and ubiquitous fatty acids at 0.1 ppm. The spectra were acquired with a time-domain of 4 megawords and 400 scans were accumulated within a mass range of *m*/*z* 92 to 1000. A routine resolving power of 400,000 at *m*/*z* 300 was achieved [14,19].

### 3.4. Processing of FT-ICR-MS Data

Raw spectra were post-processed using the software Compass DataAnalysis 4.2 (Bruker Daltonics, Bremen, Germany). Peaks processing was very conservative with a signal-to-noise ratio (S/N) of at least 4 that were exported to mass lists [19]. For all samples, exported *m*/*z* features were aligned into a matrix containing averaged *m*/*z* values (peak alignment window width: ±1 ppm) and corresponding peak intensities. Molecular formulae were assigned to the exact *m*/*z* values by mass difference network analysis using an in-house developed software tool NetCalc [41]. In total, the matrix containing the entire sample set presented 506 detected features that could be assigned to distinct and unique molecular formulae. More than 90% of all assignments were found within an error range lower than 0.2 ppm. All further calculations and filtering were done in Perseus 1.5.1.6 (Max Planck Institute of Biochemistry, Martinsried, Germany) and R Statistical Language (version 3.1.1). To validate the detection of a mass for a given condition (for example SBT or BBAI), a given mass had to be detected in at least 2 of the 3 replicates, which, in consequence, left only 307 masses in total. Annotation of formulae was made using the METLIN database and assignation to metabolic pathways was performed using MASSTRIX database coupled with KEGG Color Mapper tool.

### 3.5. Repeatability of FT-ICR-MS Measurements

Quality control (QC) samples were produced by mixing all extract samples in equal amounts. To monitor the reproducibility of the measurements overtime, QC samples were injected at the beginning and after every 10 samples (Figure S1). The coefficient of variation was calculated from the peak intensities of all elemental compositions detected in the QC samples (Figure S1D). More than 90% of all elemental compositions showed a CV-value lower than 20%.

### 3.6. Statistical Analysis

Treatment of mass lists and analysis of variance (ANOVA) with α = 0.05 were performed using Perseus 1.5.1.6 (Max Planck Institute of Biochemistry, Martinsried, Germany). Principle component analysis (PCA) and hierarchical clustering analysis (HCA) were performed using R software (version 4.0.1). van Krevelen diagrams (O/C versus H/C elemental ratios) and multidimensional stoichiometric compounds classification (MSCC) have been used to elucidate main compound categories commonly defined as lipids, peptides, amino acids, carbohydrates, nucleotides and polyphenols compounds [15,17]. Venn diagrams were generated using MOLBIOTOOLS’ Multiple List Comparator (https://www.molbiotools.com/listcompare.php, accessed on 2 August 2021).

## 4. Conclusions

Exo-metabolomes of kombucha microorganisms grown in sugared black tea in monocultures and cocultures allowed to highlight new hypotheses regarding microbial interactions that could occur during kombucha production. Interaction between the two major yeast species *B. bruxellensis* and *H. valbyensis* of the original kombucha culture induced an increase in molecular diversity and so did the yeast–acetic acid bacterium pairing *B. bruxellensis* and *A. indonesiensis*. Oppositely, the interactions that occurred in cocultures involving *H. valbyensis* and *A. indonesiensis*, with or without the presence of *B. bruxellensis* (HVAI and T), induced lower molecular diversity. Those phenomena explain the dendrogram shown in Figure 1A, because except when all three microorganisms where together, *B. bruxellensis* influenced greatly the metabolomes in BBAI and BBHV (but not T). To a lesser extent, the presence of *H. valbyensis* with *A. indonesiensis* did not bring as many changes compared to the acetic acid bacterium monoculture. Finally, the interaction of all three microorganisms appeared to cancel the effects of the other interactions (in particular those from the yeast–yeast interaction), thus bringing the exo-metabolome closer to sugared tea and the monocultures. Polyphenols and phytochemicals were widely affected by microbial interactions. Nevertheless, it can also be supposed that microbial interactions generated different compounds and physical chemical conditions that could interact and modify plant secondary metabolites in different ways. Moreover, it has been demonstrated that polyphenols could be absorbed by yeast cell wall [42]. However, detailed interpretation of the most abundant metabolites analyzed with FT-ICR/MS raised new hypotheses regarding microbial activities and metabolites from different molecular families: fatty acids, peptides, and carbohydrates. It highlighted potential recurring commensalism between yeasts and the acetic acid bacterium through hydroxy stearic acid (released from yeasts and consumed by the acetic acid bacterium) or dehydroquinic acid (released from the acetic acid bacterium and consumed by the yeasts). Such exchanges of metabolites point toward mutualistic interactions that characterize stable communities of organisms. By taking support from these results, further investigation on less abundant metabolites and using targeted analyses could confirm the existence of such interactions with a focus on specific novel compounds and pathways. Moreover, acetic acid bacteria remain unproperly studied. Similar metabolomic approaches using non-targeted analytic tools could bring significant advances in the understanding of interspecies interactions for this group of bacteria, in the context of kombucha, for example.

## Data Availability

The data presented in this study are available within the article and supplementary data.

## Supplementary Materials

The following supporting information can be downloaded at: https://www.mdpi.com/article/10.3390/metabo12030235/s1, Figure S1: Visualization of Quality control (QC) samples. (A) maximum, (B) average and (C) minimal ion intensities measured in QC samples. (D) Distribution of mass number according to variation coefficient of QC samples. (E) Principal Component Analysis d1 coordinate of samples according to passage number, with QC samples signalized in red; Table S1: Database annotation of markers; Figure S2: Distribution of annotated compounds using MASSTRIX database according to metabolic pathways according to KEGG Mapper Color; Figure S3: Venn diagram showing the number of common and unique formulae between those produced in BBHV as part of the interaction between *B. bruxellensis* (BB) and *H. valbyensis* (HV) (labeled “BBHV(BBHV)” and those present in BBHV but inhibited by the presence of *A. indonesiensis* (AI) in the coculture gathering all three microorganisms (T) (labeled “BBHV(T)”); Figure S4: Venn diagram showing the number of common and unique formulae between the lists of those associated with *A. indonesiensis* monoculture (AI), that were negatively impacted by the presence of yeast(s). The three cases involved *B. bruxellensis* alone (BBAI), *H. valbyensis* alone (HVAI) and the two yeasts simultaneously (T).
