# Supplementary material for: Microbial Interactions in Kombucha through the Lens of Metabolomics
Source: Metabolites. 2022 Mar 9;12(3):235. doi: 10.3390/metabo12030235 (PMC8954749; doi:10.3390/metabo12030235)

## Supplementary Material

# Microbial Interactions in Kombucha through the Lens of Metabolomics

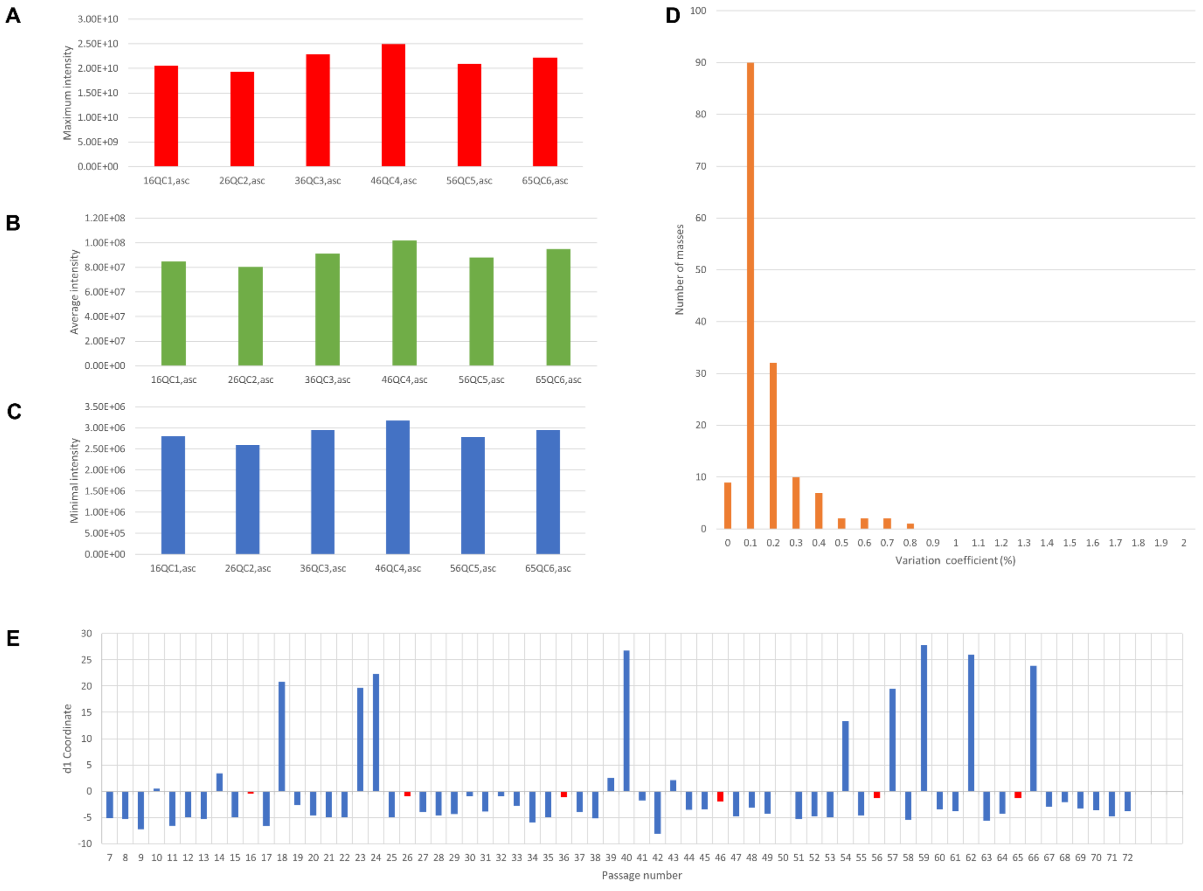


**Figure S1: Visualization of Quality control (QC) samples. (A) maximum, (B) average and (C) minimal ion intensities measured in QC samples. (D) Distribution of mass number according to variation coefficient of QC samples. (E) Principal Component Analysis d1 coordinate of samples according to passage number, with QC samples signalized in red.**

**Table S1: Database annotation of markers**

| Mass (average) | Formula | Database annotation |
|---|---|---|
| 163.04006 | $C_9H_8O_3$ | Phenylpyruvate |
| 169.01427 | $C_7H_6O_5$ | Gallic acid |
| 173.00917 | $C_6H_6O_6$ | aconitic acid |
| 175.06121 | $C_7H_{12}O_5$ | Isopropyl malate |
| 178.04774 | $C_{10}H_6O_6$ | Gluconolactone |
| 179.05612 | $C_6H_{12}O_6$ | Glucose or Fructose |
| 189.04046 | $C_7H_{10}O_6$ | Dehydroquinic acid |
| 191.01973 | $C_6H_8O_7$ | Citric acid |
| 195.05103 | $C_6H_{12}O_7$ | Gluconic acid |
| 253.21727 | $C_{16}H_{30}O_2$ | Palmitoleic acid |
| 255.23292 | $C_{16}H_{32}O_2$ | Palmitic acid |
| 281.24859 | $C_{18}H_{34}O_2$ | Oleic acid |
| 283.26424 | $C_{18}H_{36}O_2$ | Stearic acid |
| 289.07175 | $C_{15}H_{14}O_6$ | Epicatechin |
| 300.26645 | $C_{18}H_{36}O_3$ | Hydroxystearic acid |
| 341.10891 | $C_{12}H_{22}O_{11}$ | Sucrose |
| 441.08278 | $C_{22}H_{18}O_{10}$ | Epicatechin gallate |
| 503.16189 | $C_{18}H_{32}O_{16}$ | Dextrin |
| 535.15180 | $C_{18}H_{32}O_{18}$ | 1,4-bêta-D-Glucan |

**Figure S2: Distribution of annotated compounds using MASSTRIX database according to metabolic pathways according to KEGG Mapper Color.**

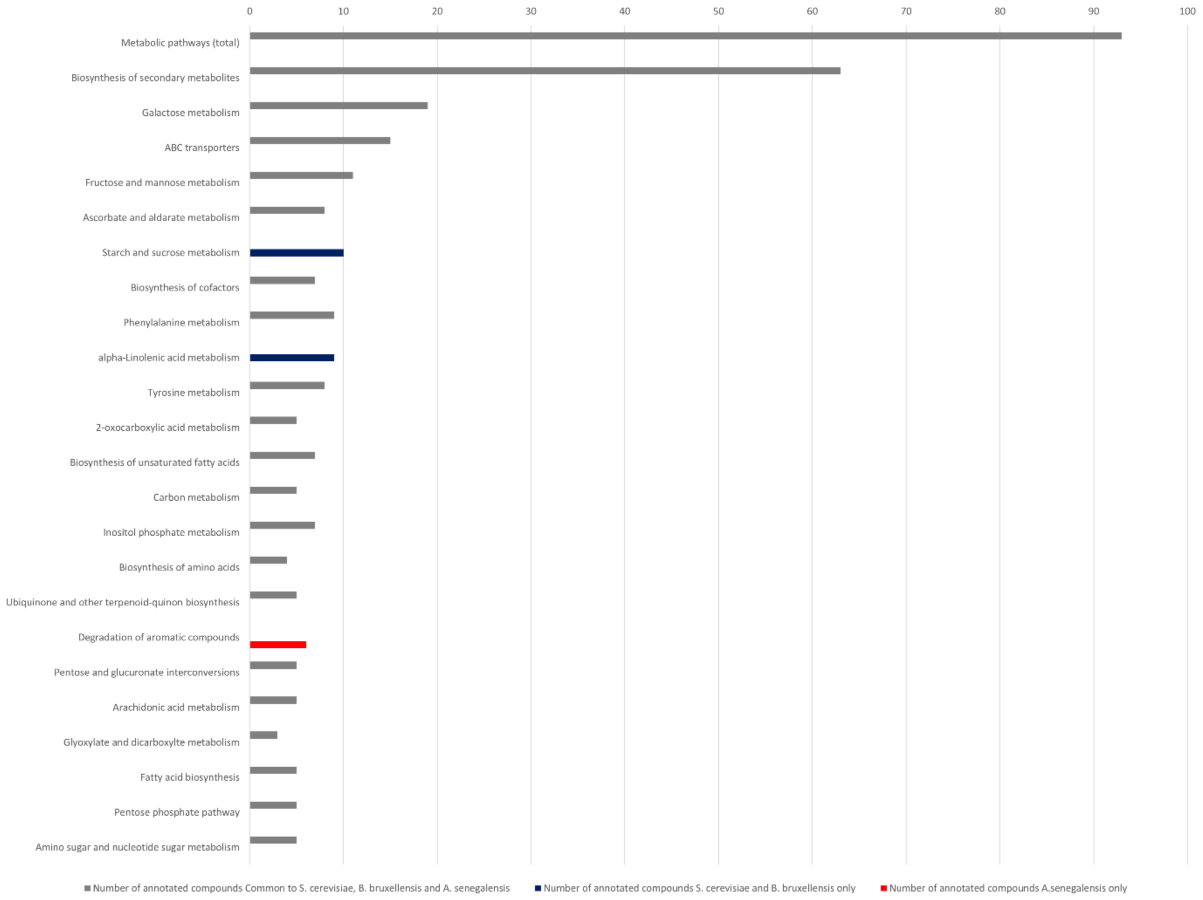

**Figure S3: Venn diagram showing the number of common and unique formulae between those produced in BBHV as part of the interaction between *B. bruxellensis* (BB) and *H. valbyensis* (HV) (labeled "BBHV(BBHV)" and those present in BBHV but inhibited by the presence of *A. indonesiensis* (AI) in the coculture gathering all three microorganisms (T) (labeled "BBHV(T)").**

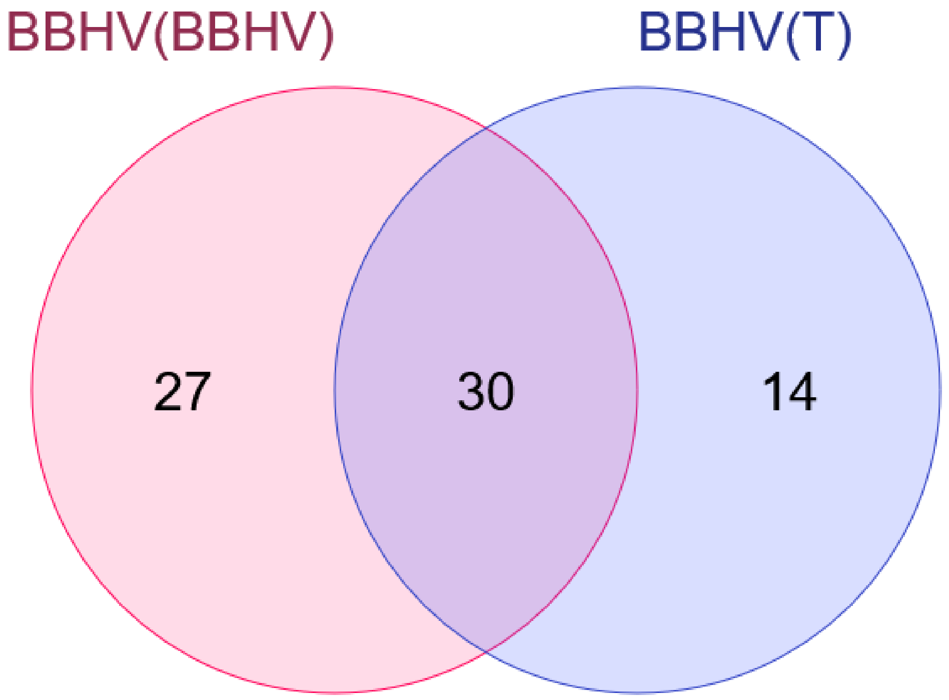

**Figure S4: Venn diagram showing the number of common and unique formulae between the lists of those associated with *A. indonesiensis* monoculture (AI), that were negatively impacted by the presence of yeast(s). The three cases involved *B. bruxellensis* alone (BBAI), *H. valbyensis* alone (HVAI) and the two yeasts simultaneously (T).**

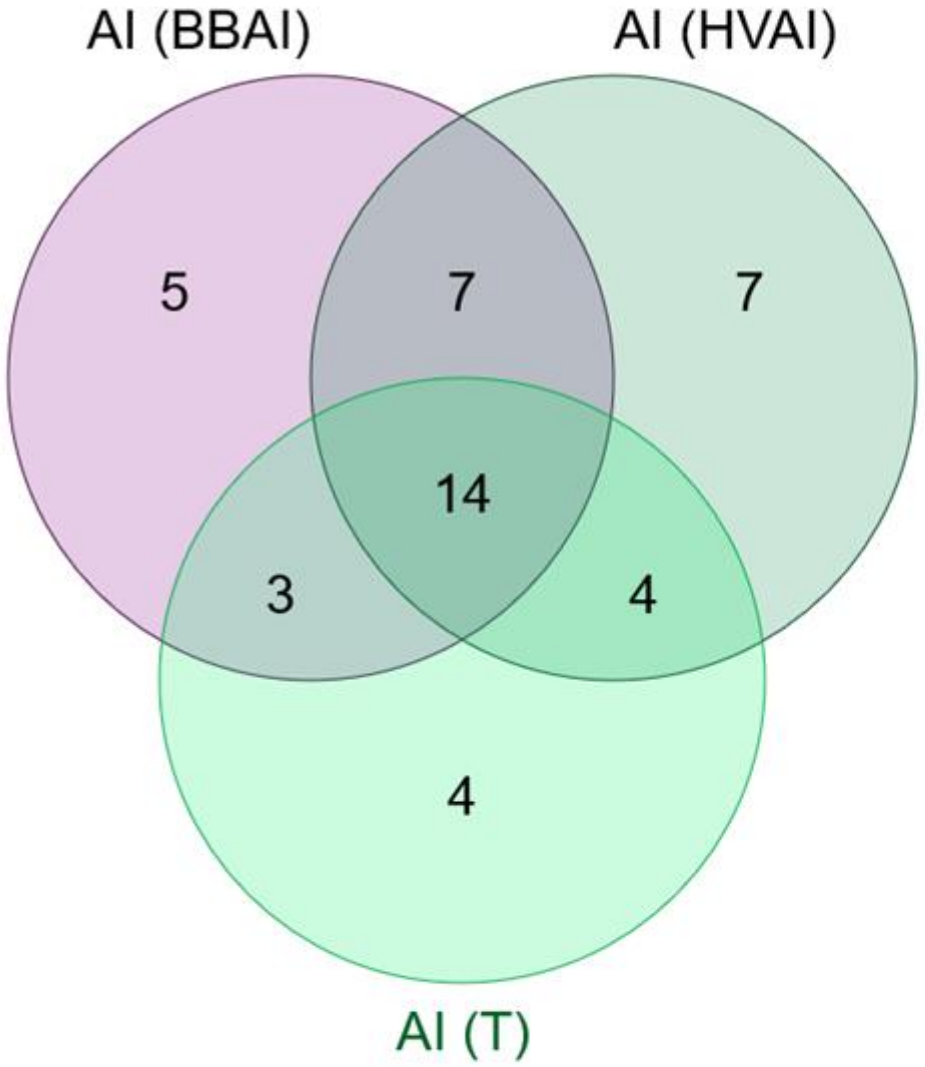

Supplement: Supplementary file 1 [file metabolites-12-00235-s001.zip › metabolites-1575371-supplementary.pdf]
